# Spontaneous spondylodiscitis and endocarditis: interdisciplinary experience from a tertiary institutional case series and proposal of a treatment algorithm

**DOI:** 10.1007/s10143-021-01640-z

**Published:** 2021-09-11

**Authors:** Lennart Viezens, Marc Dreimann, André Strahl, Annika Heuer, Leon-Gordian Koepke, Benjamin Bay, Christoph Waldeyer, Martin Stangenberg

**Affiliations:** 1 Division of Spine Surgery, Department of Trauma and Orthopedic Surgery, University Medical Center Hamburg-Eppendorf, Martinistr. 52, Hamburg, Germany; 2 Division of Orthopedics, Department of Trauma and Orthopedic Surgery, University Medical Center Hamburg-Eppendorf, Hamburg, Germany; 3 Department of Cardiology, University Heart and Vascular Center Hamburg, University Medical Center Hamburg-Eppendorf, Hamburg, Germany

**Keywords:** Spondylodiscitis, Endocarditis, Spinal infection, Treatment algorithm

## Abstract

Previously, the simultaneous presence of endocarditis (IE) has been reported in 3–30% of spondylodiscitis cases. The specific implications on therapy and outcome of a simultaneous presence of both diseases are not yet fully evaluated. Therefore, the aim of this study was to investigate the influence of a simultaneously present endocarditis on the course of therapy and outcome of spondylodiscitis. A prospective database analysis of 328 patients diagnosed with spontaneous spondylodiscitis (S) using statistical analysis with propensity score matching was conducted. Thirty-six patients (11.0%) were diagnosed with concurrent endocarditis (SIE) by means of transoesophageal echocardiography. In our cohort, the average age was 65.82 ± 4.12 years and 64.9% of patients were male. The incidence of prior cardiac or renal disease was significantly higher in the SIE group (coronary heart disease SIE *n* = 13/36 vs. S *n* = 57/292, *p* < 0.05 and chronic heart failure *n* = 11/36 vs. S *n* = 41/292, *p* < 0.05, chronic renal failure SIE *n* = 14/36 vs. S *n* = 55/292, *p* < 0.05). Complex interdisciplinary coordination and diagnostics lead to a significant delay in surgical intervention (S = 4.5 ± 4.5 days vs. SIE = 8.9 ± 9.5 days, *p* < 0.05). Mortality did not show statistically significant differences: S (13.4%) and SIE (19.1%). Time to diagnosis and treatment is a key to efficient treatment and patient safety. In order to counteract delayed therapy, we developed a novel therapy algorithm based on the analysis of treatment processes of the SIE group. We propose a clear therapy pathway to avoid frequently observed pitfalls and delays in diagnosis to improve patient care and outcome.

## Introduction

The incidence of spondylodiscitis is increasing in the western world [11, 12]. Amongst other things, this is the result from an increasing life expectancy which is associated with increased multimorbidity including a wide variety of pre-existing conditions and accordingly negative impact on the immune system [21]. Spontaneous spondylodiscitis can present itself in various clinical manifestations which require different therapeutic measures from conservative to surgical treatment. Even though previous retrospective studies analyzed specific indications and corresponding outcome, there is still no gold standard regarding therapy pathways [28]. It has previously been reported that a concomitant infective endocarditis (SIE) is present in up to 30% of spondylodiscitis cases [3, 6, 7, 24]. Endocarditis (IE), like spondylodiscitis, is a life-threatening disease with a mortality rate of up to 30% [14, 23, 29] and also shows an increasing incidence due to demographic changes [9].

The simultaneous occurrence of such serious diseases has rarely been described and analyzed previously. Also, the frequency of IE in spondylodiscitis cohorts has not yet been sufficiently investigated and reported rates differ greatly from 4–10% [6, 24] to 30% [3]. Confirmation of diagnosis is greatly influenced by the screening method used [3, 24]. Behmanesh et al. were able to show that the rate of diagnosed IE was 10 times higher after implementing routine screening using transoesophageal echocardiography (TOE) in patients with known spondylodiscitis [3, 6, 20].

Patients with known IE presenting with even mild symptoms of back pain should undergo an in-depth diagnostic workup using MRI (magnetic resonance imaging) to avoid missed or delayed diagnosis of spondylodiscitis [6]. Especially in an older population, delayed diagnosis of spondylodiscitis is associated with increased morbidity and mortality rates of up to 27% [6, 9–12].

The utilization of TOE as imaging method has been integrated as an essential part of treatment pathways for patients with spondylodiscitis [3, 6, 20] and is routinely performed where previous transthoracic echocardiography (TTE) has proven inconclusive.

From an interdisciplinary viewpoint of the treating team of physicians including spinal surgeons, cardiologists, cardiac surgeons, and infectious disease specialists, the relevance of SIE regarding therapeutic strategies and outcome is unclear. Previous studies have reported increased mortality rates by up to 10 times in case of SIE [3]. So far, neither an optimal surgical strategy nor definite anti-infective guidelines have been established in this subpopulation [28]. Thus, standardized treatment algorithms or recommendations for patients presenting with SIE are lacking. It has been established that for both spondylodiscitis and IE, time to diagnosis has a significant influence on prognosis and mortality [13, 14, 16, 18].

We therefore aimed to use a prospectively managed database to analyze the influence of a simultaneously present IE in patients with already diagnosed spondylodiscitis in regard to differences in clinical care. We furthermore aimed to develop a novel treatment algorithm to be able to standardize diagnostic as well as therapeutic strategies in this cohort of patients.

## Material and methods

### Study population and data procurement

The study was carried out in accordance with the Helsinki Declaration. A positive vote was received by the responsible ethics committee (medical chamber, WF-013/20). Regarding the anonymized data, there was no further consultation by the ethics committee and no informed consent was necessary.

**Fig. 1:**
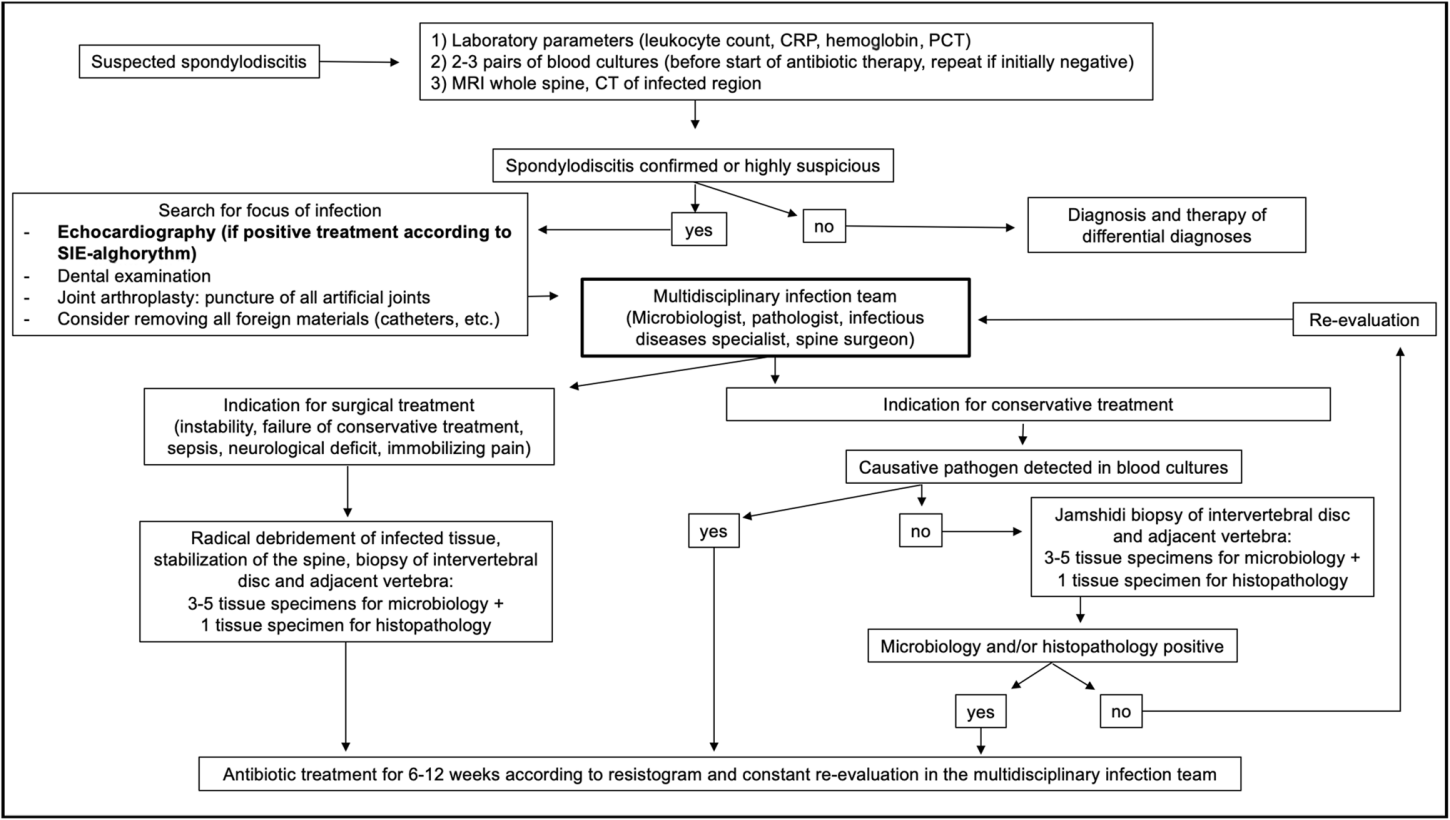
Standard spontaneous spondylodiscitis treatment algorithm at the author’s institution. All patients endorsed in this study were treated according to this algorithm

In an 8-year period from January 1, 2013, to December 31, 2020, all patients treated with proven spontaneous spondylodiscitis at a tertiary centre were included in a prospective database. All patients were treated, after the suspicion of spontaneous spondylodiscitis was raised, according to our internal algorithm, which is shown in Fig. 1. An analysis of this database was carried out in January 2021. All patients with complete documentation were included in the evaluation.

**Fig. 2:**
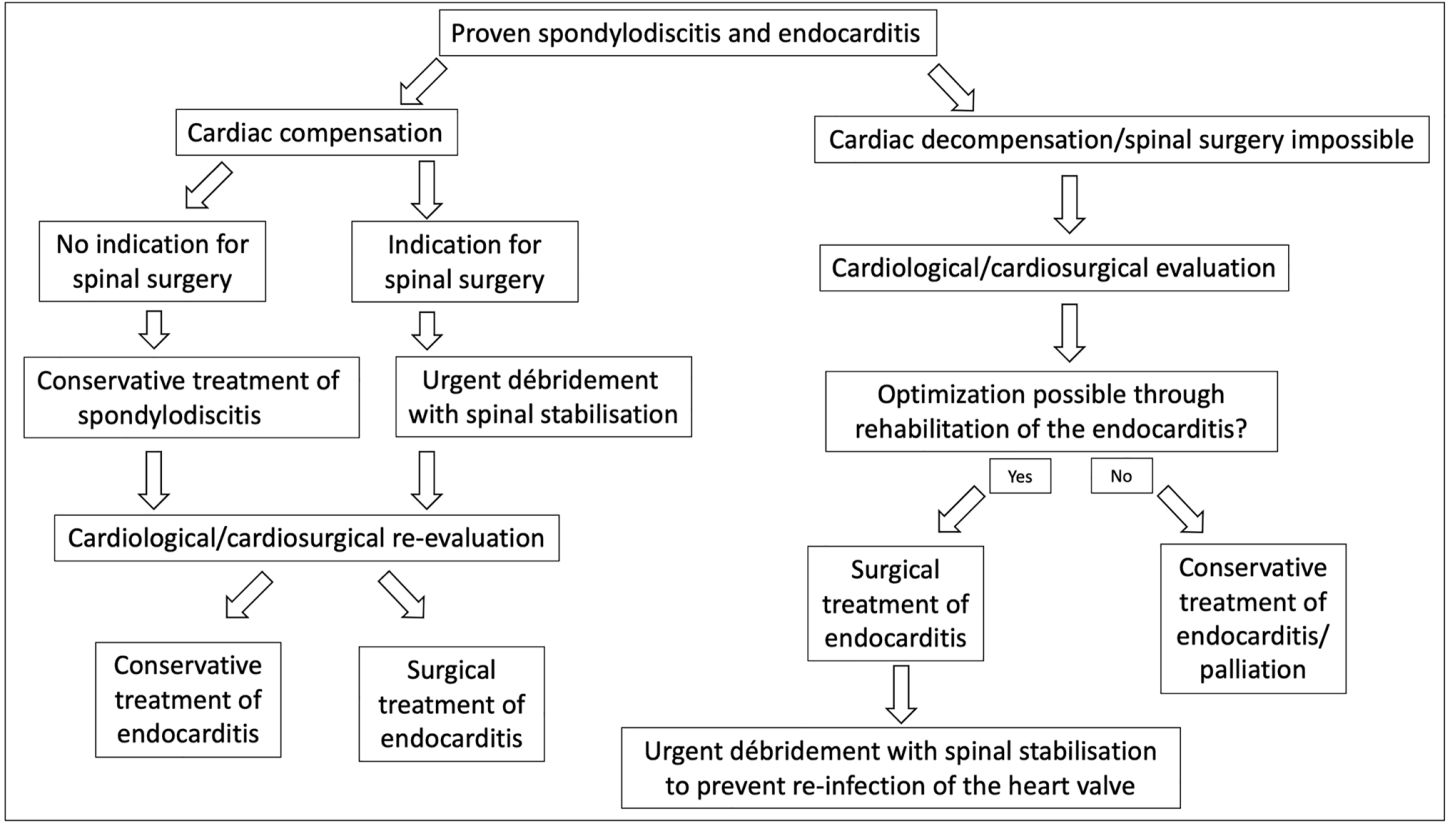
Distribution of infected valves (*n*) diagnosed with echocardiography in patients with SIE

Demographic and disease-relevant patient data were collected in all cases. All pre-existing conditions were documented and sorted into the groups listed in Table 1. Pathogens detected via microbiological screening of intraoperative tissue samples or blood cultures were evaluated. SIE was diagnosed via echocardiography at initial presentation and follow-up screenings if necessary. In addition, the duration from admission to spinal surgery and/or surgical valve replacement/repair as well as all complications until discharge were recorded. Therapeutic procedures and diagnostic steps were analyzed according to a modular principle to build a novel, optimized treatment algorithm, which is shown in Fig. 2.

**Table 1:** Overview of demographics, distribution of infected segments, and secondary diagnoses (values are given as total number (percent))

Baseline characteristics		
Variables		Characteristics
Age (years)		65.8 ± 14.1
Gender	Female	115 (35.1%)
Localization	Cervical	38 (11.6%)
	Thoracic	80 (24.4%)
	Lumbar	180 (54.9%)
	Disseminated	30 (9.1%)
Secondary diagnosis	Malignoma	77 (23.5%)
	Multidrug-resistant bacteria colonization	71 (21.6%)
	Coronary heart disease	70 (21.3%)
	Chronic renal failure	69 (21%)
	Diabetes mellitus	63 (19.2%)
	Obesity (body mass index ≥ 30 kg/m^2^)	58 (17.7%)
	Coronary heart failure	52 (15.9%)
	COPD	46 (14%)
	Chronic alcohol or i.v. drug abuse	46 (14%)
	Rheumatoid arthritis	23 (7.0%)
	Hepatitis B/C	18 (5.5%)
	Post stroke	18 (5.5%)
	Liver cirrhosis	17 (5.2%)
	Chronic urinary tract infection	17 (5.2%)
	Osteoporosis	14 (4.3%)
	Dialysis	9 (2.7%)
	Post organ transplant	8 (2.4%)
	HIV	5 (1.5%)
	Parkinson’s disease	5 (1.5%)

### Statistical analysis

The statistical analysis was performed using SPSS version 25 (IBM, New York, USA). Continuous variables are expressed as mean ± standard deviation (SD) whilst categorical variables are expressed as number (%). To compare patients with and without endocarditis in terms of continuous variables, the Student *t*-test for independent samples was used for normally distributed data. Mann–Whitney *U* test was used with non-normally distributed data, and chi^2^ or Fisher’s exact test was calculated for categorial variables. *p*-values below 0.05 were considered to indicate statistically significant differences between the two groups. Kolmogorov–Smirnov test was used to check data for normal distribution. An additional propensity score matching (PSM) was then carried out in order to achieve better comparability in a retrospective evaluation between the groups. The PSM procedure was conducted with R essentials plug-in for SPSS. To minimize the selection bias, a 1:1 ratio PSM with the “nearest neighbour matching” algorithm was performed. The score is derived from a logit model considering age and gender as predictors. The estimated PSM was then used to construct the age and gender-matched comparison groups (Table 2).

**Table 2:** Overview of recorded complications in comparison of S end SIE after PSM of 36 pairs

Complications after PSM			
Complication	S (*n*)	SIE (*n*)	*p*-value
Acute renal failure	11	13	0.62
Myocardial infarction	0	1	0.31
Cardiac decompensation	6	5	0.74
Stroke	1	3	0.3
Pneumonia	7	3	0.17
Acute liver failure	1	1	1
Delirium	7	10	0.41
Neurological deterioration	1	0	0.31
Postoperative atrial fibrillation	3	2	0.64

## Results

**Fig. 3:**
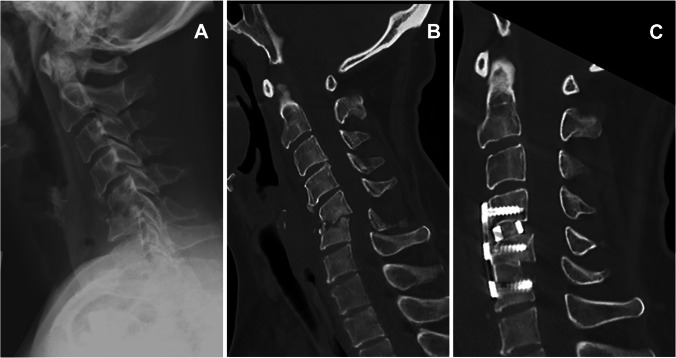
Distribution of causative pathogens found in patients with SIE

Three hundred twenty-eight consecutive cases of spontaneous spondylodiscitis were included in this analysis. The mean age was 65.82 ± 4.12 years and 213 (64.9%) patients were male. On average, imaging showed spondylodiscitis in 1.5 segments (1–7 segments). Most commonly, a lumbar (54.9%), followed by thoracic (24.4%) and cervical (11.6%), manifestation was diagnosed. In 9.1% of cases, dissemination in multiple sections of the spine was present. On average, the patients had 2.4 ± 1.9 pre-existing conditions. An overview of pre-existing conditions and demographics is listed in Table 1. If no pathogen could be detected in blood cultures, a transpedicular Jamshidi biopsy under general anaesthesia of the affected intervertebral disc space was carried out for further microbiological and histopathological analysis. In our experience, a percutaneous biopsy via the radiology colleagues is very painful for the patients and often brings too little material to perform an adequate microbiological and histopathological examination. Table 3 shows the percentage of pathogen detection in blood culture and biopsy. Once the pathogen was identified, the antibiotic treatment was adapted to its antimicrobial susceptibility pattern. Time of intravenous treatment period and oralization thereafter was discussed at a weekly interdisciplinary conference including microbiologists, infectious disease specialists, pathologists and spinal surgeons (Fig. 1). A total of 43 (13.1%) patients received conservative therapy only. The decision whether surgical therapy was necessary was made in interdisciplinary discussion depending on the patient’s clinical condition and responsiveness to antibiotic therapy. In patients with a septic deterioration, neurological deficits, severe spinal instability, deformity caused by the infection, or a significant abscess detected on the MRI, indication for spinal surgical treatment was given. In general, conservative therapy was initially attempted in all patients after MRI imaging of the entire spine and CT of the infected area. Exceptions to this were epidural abscesses with relevant spinal compression and accompanying neurological deficits or a septic clinical condition that required intensive care therapy. If, in the case of severe instability, as shown in Fig. 3, persistent pain occurred despite antibiotic therapy in accordance with the antibiogram, surgical stabilization was recommended.

**Table 3:** Overview of pathogen detection in blood culture or intraoperative biopsy

Pathogen detection	S *n* (%)	SIE *n* (%)	*p*-value
Blood culture	133/260 (51.2)	30/35 (85.7)	< 0.001
Intra-OP biopsy	192/272 (70.6)	16/31 (51.6)	< 0.05

Epidural abscesses were present in 53% of patients and psoas abscesses in 27.1% cases. The indication for surgical therapy of the IE was set in interdisciplinary discussion within the endocarditis team according to current guideline recommendations [15].

**Fig. 4:**
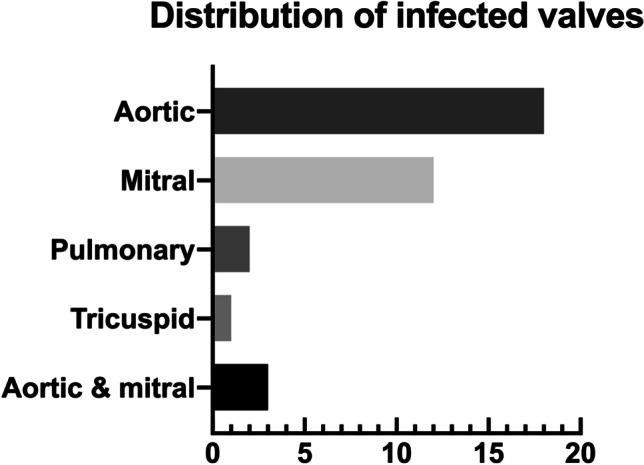
Example of severe spinal instability with subluxation, osseous destruction, and consecutive kyphosis due to cervical spondylodiscitis. **A** Sagittal x-ray. **B** Preoperative sagittal CT. **C** Postoperative sagittal CT

In 36/328 (11.0%) patients, IE was diagnosed via echocardiography. Only six performed transthoracic echocardiographies (TTE) proved a definite IE; in the other patients, TOE was used to screen for IE. The aortic valve (50.0%), followed by the mitral valve (33.3%), was the most commonly affected heart valve (Fig. 4).

Patients in the SIE group had significantly higher rates of previous cardiac diseases (coronary heart disease SIE *n* = 13 vs. S *n* = 57, *p* = 0.02; chronic heart failure SIE *n* = 11 vs. S *n* = 41, *p* = 0.01) or chronic renal failure (SIE *n* = 14 vs. S *n* = 55, *p* < 0.01).

**Fig. 5:**
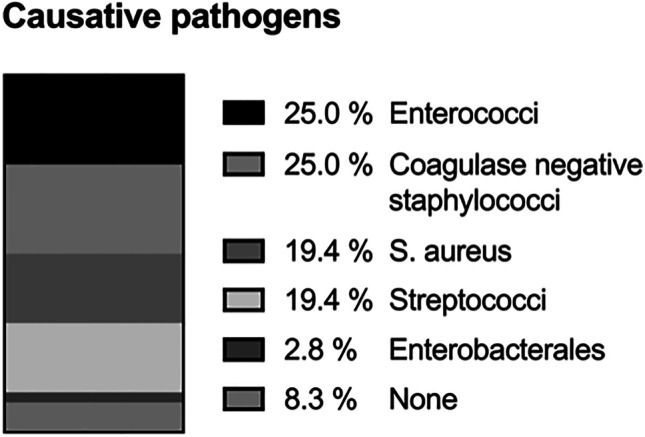
Proposed treatment algorithm derived from the analyses of all SIE cases in this study to accelerate the process of decision-making in patients with concomitant SIE in the future

In case of diagnosed SIE, microbiological results showed coagulase negative Staphylococci (CNS) and Enterococcus species to be the most common pathogens (Fig. 5). In the SIE group, 86.1% (31/36) of blood cultures were positive, whereas in the S group, only 45.6% (133/292) were positive (*p* < 0.001).

In the group of patients with SIE, the male sex was predominant (69.4%). There was no difference in the distribution of localizations between different patient groups (i.e. cervical, thoracic, lumbar). The rate of positive blood cultures was significantly increased in the SIE group (51.2% vs. 85.7%, *p* < 0.001).

In the SIE group, surgical treatment of spondylodiscitis was carried out in 28/36 (77.8%) cases whilst IE was treated surgically in 16/36 (44.4%) cases.

The in-hospital mortality overall was 14.0% (*n* = 46). In the S group, 13.4% (*n* = 39) died, whilst in the SIE group, 19.4% (*n* = 7; *p* = 0.32) died. Of those patients who died in the SIE group, all patients were treated conservatively in regard to their endocarditis (*p* = 0.01).

PSM was used to counteract biases caused by a relatively small sample size and age as major factor for mortality. Thirty-six pairs could be formed and showed no significant difference in mortality (S: *n* = 4, SIE: *n* = 7, *p* = 0.33).

Time from admission to surgical treatment almost doubled in the SIE compared to the S group (S = 4.5 ± 4.5 days vs. SIE = 8.9 ± 9.5 days, *p* < 0.05). Furthermore, intravenous anti-infective treatment in the SIE group was delayed (S = 26.3 ± 21.3 vs. SIE = 43.9 ± 21.9 days; *p* > 0.05) which resulted in a prolonged in-hospital stay (S = 27 ± 23 vs. SIE = 34 ± 21 days; *p* > 0.05. After performing PSM, no significant changes within the complication rates were observed (Table 2). After a case by case analysis, 35 patients could be grouped and divided onto 5 different diagnosis-treatment pathways. Only one patient who received surgical therapy for IE with following conservative therapy for spondylodiscitis cannot be found in the treatment algorithm developed (Fig. 2). In the one case, which could not be included in our treatment algorithm, the initial MRI showed a mild spondylodiscitis and the patient rejected surgical therapy despite the interdisciplinary advice because he was pain free.

## Discussion

In this study, we analyzed the influence of concomitant endocarditis on therapy and outcome in the largest group of patients with spondylodiscitis published to date. A total of 328 patients with spondylodiscitis were examined over an 8-year period using a well-established interdisciplinary approach. IE was diagnosed in 36 cases. As previously described by various authors, there is a predominance of the male sex in both the S and SIE group [6, 8, 9, 27]. Age and gender distribution in our collective was similar to those of other large spondylodiscitis [10] and endocarditis [6] collectives. Patients with IE showed typical distributions of affected valves [26]. Localization of spondylodiscitis did not differ between the S and SIE groups and coincide with literature findings [19]. In our collective, echocardiography was performed in 77.5% of patients in order to rule out the presence of IE, resulting in 36 patients diagnosed with SIE. This corresponds to a rate of 11.0% overall or 14.1% by means of conducted echocardiographic screening. This is significantly less than Pigrau et al. and Behmanesh et al., who were able to detect concomitant IE in 30% and 32% of spondylodiscitis cases [3, 24]. Nevertheless, our study shows that IE is not uncommon in patients presenting with spontaneous spondylodiscitis and therefore echocardiography should be a set step in any diagnostic algorithm used in clinical practice [20]. In our patient collective, the diagnosis was only possible in six cases by means of TTE. Despite the fact that TOE is more invasive and more expensive than TTE, we strongly recommend performing this diagnostic step as it proves a significantly higher sensitivity than a TTE [2, 25]. This recommendation coincides with the corresponding recommendations of cardiological and orthopaedic societies [4, 14, 17, 31]. We observed a significantly higher rate of positive blood cultures in the SIE group (Table 3) which is explained by the valvular localization of an infectious focus where bacteriaemia is more frequent [22]. In contrast, intraoperative samples in the SIE group showed less frequent detection of pathogens (70.6% vs. 51.6%, *n* < 0.05). We attribute this to the fact that, due to the early positive blood cultures in the SIE group, we had already frequently started preoperative antibiotic therapy, which made intraoperative bacterial detection more difficult [1].

Known risk factors for the development of IE [6] include pre-existing cardiac diseases such as CHD and CHF as well as chronic renal failure. Pre-existing cardiac and renal conditions proved significantly more frequent in the SIE than in the S group.

Compared to previous studies, we demonstrated a low rate of conservative therapy [5, 30], which is likely due to our spine centre being part of a tertiary hospital where many complex cases are referred to. For example, 23/36 patients with IE were referred to us from primary hospitals where conservative treatment had failed, or due to the complexity of the case. Furthermore, in cases of SIE, a surgical debridement and stabilization are mandatory after surgical valve replacement to prevent for early reinfection as it is shown in our treatment algorithm (Fig. 2).

With this study, we were able to show for the first time that concomitant spondylodiscitis and infective endocarditis diagnosed by echocardiography has a significant consequence regarding course of treatment compared to patients only diagnosed with spondylodiscitis. Anti-infective therapy was almost administered twice as long intravenously to the patients in the SIE group than to patients in the S group. Accordingly, the length of the in-hospital stay was also increased in the SIE group. Furthermore, we were able to show a significant delay of surgical therapy if IE was diagnosed. As frequently described in literature [13, 14, 16, 18], this may lead to poorer overall outcomes.

Analyzing each patient’s treatment pathway endorsed in this study, the interdisciplinary coordination and decision-making process proved to be the most likely cause of this observed time loss and treatment delay in the SIE group. In avoidance of such obstacles, the flowchart shown in Fig. 2 was developed in order to provide a ready-to-use therapy sequence which aims towards a more efficient coordination effort within the interdisciplinary team. In our department, it was put in place as a result of this study in December 2020.

### Limitations

The limitations of this study result from its retrospective and monocentric design. Furthermore, it should be mentioned that all patients were treated at a university hospital and, as already mentioned, a high number of externally transferred patients with failed conservative therapy were included so it must be stated that successful conservative therapy of spondylodiscitis is certainly underrepresented in our cohort.

## Conclusion

For the first time, this study was able to show that a simultaneous presence of IE in patients with an already diagnosed spondylodiscitis does not lead to increased mortality in a large collective, but significantly increases time to surgical treatment as well as time of hospitalization. The case by case analysis showed that part of this prolonged diagnosis and treatment process resulted from preoperative interdisciplinary discussions concerning therapeutic options. In order to avoid delayed treatment, this study presents an algorithm which should be implemented to safely accelerate the process of decision-making and subsequently improving patient outcome.

## Data Availability

All relevant data are included in the manuscript. Additional data and information will be provided from the corresponding author upon reasonable request.

## Abbreviations

S: Spondylodiscitis
SIE: Spondylodiscitis and endocarditis
IE: Infective endocarditis
TOE: Transoesophageal echocardiography
TTE: Transthoracic echocardiography
MRI: Magnetic resonance imaging
MSSA: *Staphylococcus aureus*
CHD: Coronary heart disease
CHF: Chronic heart failure
PSM: Propensity score matching
